# Supplementary material for: Analysis of PPARγ Signaling Activity in Psoriasis
Source: Int J Mol Sci. 2021 Aug 10;22(16):8603. doi: 10.3390/ijms22168603 (PMC8395241; doi:10.3390/ijms22168603)
Supplement: Supplementary file 1 [file ijms-22-08603-s001.zip › Supplemental materials_Analysis of PPARg signaling activity in psoriasis/Pathway models/Models images and html files/PPARG inhibits phenotypes assotiated with psoriasis/30692.html]

virus infection


# Disease virus infection

|  |  |
| --- | --- |
| URN | urn:agi-meshdis:Virus%20Diseases |
| Total Entities | 28 |
| Connectivity | 6794 |
| Name | virus infection |

---

|  |  |
| --- | --- |
| ChildConcepts | viral cytopathic effect |
|  | DNA virus infection |
|  | viral skin infection |
|  | human viral hepatitis |
|  | virus persistence |
|  | severe viral infections |
|  | recurrent virus infection |
|  | viral ear infection |
|  | viral gastroenteritis |
|  | viral pharyngitis |
|  | viral respiratory tract infection |
|  | viral sinusitis |
|  | viral upper respiratory tract infection |
|  | viral keratitis |
|  | viral myocarditis |
|  | BK virus infection |
|  | arboviral disease |
|  | RNA virus infection |
|  | viral bronchiolitis |
|  | viral conjunctivitis |
|  | viral hemorrhagic fever |
|  | viremia |
|  | viral meningitis |
|  | viral pneumonia |
|  | slow virus infection |
|  | tumor virus infection |
|  | viral ocular infection |
|  | viral encephalitis |

---

|  |  |
| --- | --- |
| Pathway | Neutrophil Degranulation via FPR1 Signaling |
|  | Hashimoto's Thyroiditis |
|  | PPARG inhibits phenotypes assotiated with psoriasis |
|  | efects selected |

---

|  |  |
| --- | --- |
| MedScan ID | 9003353 |

---

|  |  |
| --- | --- |
| Alias | viral infections |
|  | viral infectious diseases |
|  | disease caused by virus |
|  | disease, viral |
|  | viral infection |
|  | infections, viral |
|  | virus infections |
|  | viral infectious disease |
|  | viral illness |
|  | virus disease |
|  | infection, viral |
|  | virus infection |
|  | infections of virus |
|  | disease due to virus |
|  | other viral infections |
|  | viral disease |
|  | infection by the virus |
|  | infections by the virus |
|  | infection of virus |
|  | viral diseases |
|  | virus diseases |
|  | diseases, viral |
|  | Diseases, Virus |
|  | Disease, Virus |

---

|  |  |
| --- | --- |
| MeSH ID | D014777 |
|  | M0022758 |

---

|  |  |
| --- | --- |
| MeSH Heading | Virus Diseases |

---
